# The Diabetes Paradox in Aortic Aneurysms: An Unexpected Inverse Association

**DOI:** 10.3390/biomedicines14102372

**Published:** 2026-10-17

**Authors:** Paolo De Orchi, Sara Saltarocchi, Flaminia Spunticchia, Emmanouela Chourda, Angelo Pisani, Wael Saade, Mizar D’Abramo, Piera Ciaramella, Marco Totaro, Emiliano M. Cirio, Fabio Miraldi

**Affiliations:** 1 Division of Cardiac Surgery, Azienda di Rilievo Nazionale e Alta Specializzazione “G. Brotzu”, 09135 Cagliari, Italy; emilianomariacirio@aob.it; 2 Department of General Surgery, Surgical Specialties and Anesthesiology “Paride Stefanini”, Sapienza University of Rome, 00161 Rome, Italy; sara.saltarocchi@uniroma1.it (S.S.); piera.ciaramella@uniroma1.it (P.C.); 3 Cardiac Surgery Unit, Azienda Ospedaliero—Univeristaria Policlinico Umberto I, 00161 Rome, Italy; angelo.pisani@ymail.com (A.P.); marcototaro@virgilio.it (M.T.); 4 Department of Medical and Cardiovascular Sciences, Sapienza University of Rome, 00161 Rome, Italy; flaminia.spunticchia@uniroma1.it (F.S.); emmanouela.chourda@uniroma1.it (E.C.); wael.saade@uniroma1.it (W.S.); mizar.dabramo@uniroma1.it (M.D.); fabio.miraldi@uniroma1.it (F.M.)

**Keywords:** aortic disease, aortic aneurysm, diabetes mellitus, antidiabetic medications, hyperglycemia, inflammation, wall stress

## Abstract

Aortic aneurysms are associated with substantial morbidity and mortalitbecause of the risk of progressive dilation, rupture, and related acute aortic complications. Although diabetes mellitus (DM) is a major cardiovascular risk factor, epidemiological studies have consistently reported an inverse association between DM and the prevalence and progression of abdominal aortic aneurysm (AAA). More limited evidence suggests an inverse association between diabetes and thoracic aortic aneurysm. Accordingly, this review focuses primarily on AAA; where available, evidence concerning TAA and aortic dissection is discussed separately. This review examines the pathophysiological mechanisms underlying the inverse association between DM and aortic aneurysm development. Key mechanisms include the effects of hyperglycemia on extracellular matrix remodeling, particularly through modulation of matrix metalloproteinase (MMP-2 and MMP-9) activity, increased formation of advanced glycation end products (AGEs), and enhanced collagen cross-linking. Experimental and translational evidence suggests that diabetes may influence inflammatory cell states and mural neoangiogenesis, although these effects appear to be context- and model-dependent. Antidiabetic therapies, including metformin, DPP-4 inhibitors, GLP-1 receptor agonists, thiazolidinediones, sulfonylureas, and SGLT2 inhibitors, may also contribute to the observed association through glucose-lowering and/or pleiotropic vascular effects. Furthermore, diabetes-related changes in aortic wall biomechanics, including increased wall thickness and potentially altered wall stress, may contribute to aneurysm stabilization. Overall, DM is associated with a lower prevalence and slower progression of AAA, potentially through a combination of metabolic, inflammatory, structural, and treatment-related mechanisms. These observations should not be interpreted as evidence that diabetes or chronic hyperglycemia is clinically beneficial; rather, they may help identify biological pathways relevant to aneurysm development and progression.

## 1. Introduction

Aortic diseases comprise a heterogeneous group of conditions affecting the aorta and may involve the thoracic, abdominal, or thoracoabdominal segments. Among the most clinically relevant entities are aortic aneurysm and aortic dissection. An aortic aneurysm is defined as dilation of the aorta involving all three layers of the arterial wall, with a diameter at least 50% greater than that of the adjacent normal segment [1]. Aortic dissection is an acute aortic syndrome characterized by separation of the layers of the aortic wall, typically initiated by an intimal tear or, less commonly, by intramural hemorrhage. It may occur in the presence or absence of pre-existing aortic dilation and is associated with a range of predisposing conditions, including hypertension, medial degeneration, bicuspid aortic valve, and heritable connective tissue disorders [2].

Abdominal aortic aneurysm represents a substantial global health burden. A systematic review and modeling analysis estimated a global AAA prevalence of 0.92% among individuals aged 30–79 years in 2019, corresponding to approximately 35 million affected individuals worldwide, with a markedly higher prevalence in men [3]. Thoracic aortic aneurysm is less well characterized epidemiologically; a population-based meta-analysis estimated a pooled incidence of 5.3 cases per 100,000 person-years and a prevalence of approximately 0.16% [4]. Despite declining age-standardized mortality rates, the absolute global burden of aortic aneurysm remains substantial: Global Burden of Disease 2021 estimates indicate approximately 154,000 aortic aneurysm-related deaths worldwide in 2021, reflecting the combined effects of population growth and aging [5].

Several studies have identified major risk factors for aortic aneurysm disease, including male sex, smoking, coronary artery disease, peripheral arterial disease, and a family history of aortic aneurysm [6]. Diabetes mellitus (DM) is of particular interest: although it is a major risk factor for cardiovascular disease, multiple studies have reported an inverse association between DM and AAA prevalence and growth [7]. This apparent paradox is particularly notable because the vascular effects of diabetes differ across cardiovascular phenotypes: while diabetes strongly promotes atherosclerotic cardiovascular disease through endothelial dysfunction, inflammation, oxidative stress, and metabolic injury, its association with AAA development and progression appears to follow a different epidemiological pattern. A large study investigating the relationship between DM and AAA reported a mean AAA growth rate of 2.2 mm per year [8]; however, growth was on average 0.5 mm per year slower in patients with diabetes than in those without diabetes. In addition, higher hemoglobin A1c levels were associated with slower aortic diameter expansion, irrespective of diabetes status. More limited evidence also suggests an inverse association between diabetes and TAA, although the available data remain substantially less extensive than those for AAA [7,9].

The aim of this narrative review is to critically examine the epidemiological association between diabetes and aortic aneurysm disease, with particular emphasis on AAA, and to evaluate the biological mechanisms and pharmacological factors that may contribute to this paradoxical relationship. Evidence regarding TAA and aortic dissection is considered separately and interpreted cautiously because of the limited evidence base and the substantial biological differences among aortic phenotypes.

## 2. Methods—Literature Search and Selection

A narrative literature search was performed to identify relevant evidence addressing the epidemiology, pathophysiology, molecular mechanisms, and therapeutic implications of the relationship between diabetes and aortic aneurysmal disease. The literature search was conducted using PubMed/MEDLINE and Scopus and included publications from 2001 to 2026. The final literature search was performed on 19 September 2026. No language restrictions were applied. The 2001–2026 period was selected to focus on contemporary clinical and mechanistic evidence relevant to current diagnostic, therapeutic, and molecular frameworks in aortic aneurysm disease. The principal search terms comprised combinations of “diabetes”, “diabetes mellitus”, “aortic aneurysm”, “abdominal aortic aneurysm”, “thoracic aortic aneurysm”, “aortic dissection”, “aneurysm progression”, “metformin”, “antidiabetic drugs”, “SGLT2 inhibitors”, “DPP-4 inhibitors”, “GLP-1”, “inflammation”, “macrophages”, “vascular smooth muscle cells”, “advanced glycation end products”, and “oxidative stress”, together with related molecular and cellular terms. Articles were selected according to their relevance to the scope of the review, with particular emphasis on studies addressing the association between diabetes and aneurysmal disease and the mechanisms that may underlie it. Clinical and observational studies were used primarily to assess epidemiological associations and clinical outcomes, whereas translational and preclinical studies were used to investigate potential molecular and cellular mechanisms. When available, systematic reviews and meta-analyses were considered to provide an integrated assessment of the existing evidence. Preclinical findings were interpreted primarily as mechanistic evidence and were not considered equivalent to clinical evidence. Given the narrative nature of the review, no formal systematic-review protocol or quantitative risk-of-bias assessment was applied, and the evidence was synthesized qualitatively according to its study design, biological plausibility, consistency, and clinical relevance.

## 3. Pathophysiological Mechanisms

**Figure 1:**
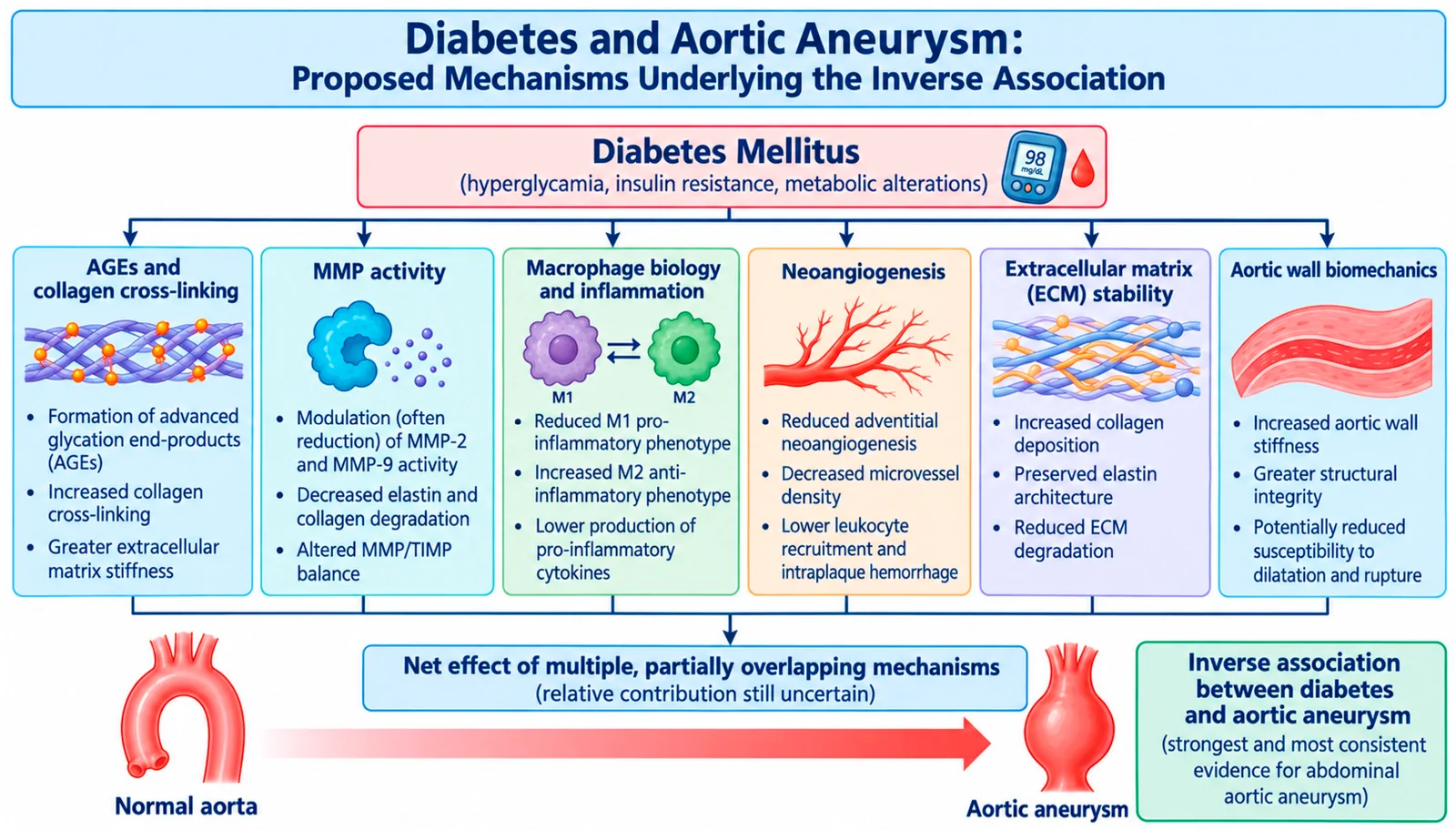
Proposed mechanisms underlying the inverse association between diabetes and aortic aneurysms.

Several pathophysiological mechanisms have been proposed to explain the inverse association between DM and AAA. Importantly, diabetes should not be considered a uniform biological exposure. The vascular effects of diabetes may differ between type 1 and type 2 diabetes and according to disease duration, degree of glycemic control, insulin resistance, insulin treatment, renal function, obesity, metabolic syndrome, and exposure to glucose-lowering therapies. Most epidemiological evidence concerning the inverse association with AAA derives from populations with type 2 diabetes, making extrapolation to other diabetic phenotypes uncertain. Accordingly, the mechanisms discussed below should be interpreted within this heterogeneous metabolic and therapeutic context [7]. These mechanisms are summarized in Figure 1.

### 3.1. Hyperglycemia

Hyperglycemia may increase collagen synthesis within the aortic extracellular matrix (ECM). MMPs are proteolytic enzymes involved in the degradation of elastin and collagen within the vascular wall, thereby contributing to extracellular matrix remodeling [6,9]. MMP-2 and MMP-9, in particular, have been implicated in aneurysm development. Human and experimental evidence suggests that diabetes and hyperglycemic conditions may modulate MMP activity, although this relationship is more complex than a uniform suppression of proteolysis. Reduced MMP secretion has been observed in activated monocytes exposed to glycated or diabetic aortic matrices, and lower circulating MMP-2 and MMP-9 concentrations have also been reported in patients with type 2 diabetes; however, other experimental conditions have produced opposite effects, indicating that the response depends on cell type, glucose concentration, and extracellular matrix context [7,10,11]. Hyperglycemia also promotes the formation of advanced glycation end products (AGEs) [6,7]. However, AGE accumulation should not be interpreted as uniformly beneficial, because AGE–RAGE signaling may simultaneously promote oxidative stress, endothelial dysfunction, inflammation, and maladaptive vascular remodeling, effects that contribute to the broader cardiovascular burden of diabetes [12,13]. Recent single-cell transcriptomic studies indicate that AAA-associated wall remodeling cannot be reduced to MMP modulation and AGE-mediated cross-linking alone. Human AAA tissue shows marked cellular heterogeneity, including depletion of contractile vascular smooth muscle cell (VSMC) states and emergence of phenotypically altered VSMC populations. Integrated human and experimental analyses have also identified VSMC senescence and impaired oxidative phosphorylation as features of aneurysmal degeneration. These observations broaden the mechanistic framework to include dynamic cell-state transitions and metabolic dysfunction within the aortic wall, although direct evidence that diabetes specifically modifies these AAA-associated VSMC programs remains limited [14,15].

### 3.2. Inflammation

Inflammation is a key component involved in aortic aneurysm development; adventitial and medial inflammatory cell infiltration has been observed in aneurysmal vessels, contributing to vascular wall remodeling and weakening [16,17]. Monocytes and macrophages represent prominent components of the inflammatory infiltrate in AAA, although their biology is considerably more heterogeneous than suggested by a simple M1/M2 classification. Circulating monocytes are conventionally divided into classical (CD14++CD16−), intermediate (CD14++CD16+), and non-classical (CD14+CD16++) subsets, and alterations in these populations have been reported in AAA. However, contemporary single-cell RNA-sequencing studies have identified multiple macrophage states characterized by overlapping inflammatory, reparative, proliferative, and interferon-responsive transcriptional programs, supporting the concept of macrophage activation as a dynamic continuum rather than a fixed M1/M2 dichotomy. Human AAA single-cell profiling has demonstrated substantial heterogeneity of both immune and vascular cell populations, whereas experimental studies have identified distinct resident, inflammatory, reparative, and interferon-inducible monocyte/macrophage subsets. These observations indicate that bulk macrophage counts or M1/M2 ratios may obscure functionally distinct cellular states. In some experimental models, hyperglycemia has been associated with reduced macrophage accumulation, MMP-9 activity, elastolysis, and neovascularization; nevertheless, these findings should be interpreted as model-specific and should not be considered evidence of uniformly suppressed inflammation in human diabetes [14,18].

Recent spatial and genomic approaches further refine this inflammatory framework. Spatially resolved transcriptomic analyses of human AAA have demonstrated region-specific inflammatory and apoptotic transcriptional programs involving macrophages, lymphocytes, and VSMCs, while single-cell spatial mapping in experimental AAA has revealed layer-specific interactions among macrophages, fibroblasts, and smooth muscle cells. In parallel, clonal hematopoiesis of indeterminate potential (CHIP) has emerged as a potential systemic inflammatory modifier of AAA risk. In a large UK Biobank analysis, CHIP was associated with incident AAA, with stronger associations observed for larger clones and ASXL1-associated CHIP; genetically reduced IL-6 signaling appeared to attenuate this excess risk. These findings suggest that AAA inflammation reflects both spatially organized local cellular networks and systemic clonal inflammatory influences. However, whether diabetes modifies these spatial or clonal immune programs remains unknown and warrants specific investigation [19,20,21].

C-peptide represents a biologically active peptide with context-dependent vascular effects that vary according to diabetic phenotype and circulating concentration. In states of C-peptide deficiency, particularly type 1 diabetes, physiological C-peptide replacement has been associated with antioxidant, anti-inflammatory, and endothelial-protective effects, including reduced ROS generation and attenuation of NF-κB activation. Conversely, elevated C-peptide concentrations, as observed in insulin resistance and early type 2 diabetes, may exert pro-atherogenic effects, including promotion of monocyte recruitment and vascular smooth muscle cell proliferation. Therefore, the vascular role of C-peptide should not be interpreted as uniformly protective, and evidence directly linking C-peptide signaling to aortic aneurysm biology remains limited [22,23].

### 3.3. Neoangiogenesis

Mural neoangiogenesis is an important feature of aortic aneurysm formation and rupture. In abdominal aortic aneurysm (AAA), this process is frequently accompanied by the formation of an intraluminal thrombus (ILT), which is not a static structure but undergoes continuous remodeling due to persistent pulsatile blood flow. Although the presence of ILT may mechanically reduce peak wall stress by increasing wall thickness, it simultaneously creates a diffusion barrier that limits oxygen transport to the underlying media. This results in localized hypoxia, which in turn promotes inflammatory cell infiltration and stimulates pathological neovascularization within the medial and adventitial layers of the aortic wall [16,24]. Experimental evidence suggests that hyperglycemia may modulate these processes. In murine models, hyperglycemic conditions have been associated with reduced aneurysm diameter, decreased medial elastin degradation (elastolysis), diminished macrophage infiltration, and attenuated neovascularization [25]. Mechanistically, hyperglycemia appears to suppress angiogenic signaling pathways, particularly through downregulation of vascular endothelial growth factor (VEGF) expression and impaired endothelial cell activation, leading to reduced neovessel formation within the aneurysmal wall. Beyond its effects on angiogenesis and inflammation, hyperglycemia may also influence aneurysm progression through additional pathways. These include modulation of the fibrinolytic system, alterations in receptor for advanced glycation end products (RAGE) signaling, and impaired function and mobilization of endothelial progenitor cells, all of which contribute to vascular remodeling and tissue repair processes [26]. Collectively, these findings suggest that hyperglycemia has a complex and somewhat paradoxical role in AAA pathobiology and may contribute to reduced aneurysm expansion through inhibition of key pathological mechanisms such as inflammation, extracellular matrix degradation, and neoangiogenesis.

### 3.4. Aortic Wall Biomechanics and Wall Stress

The aorta is exposed to greater hemodynamic stress than other arterial segments, owing to its central role in accommodating pulsatile blood flow and maintaining systemic circulation. Aortic aging is characterized by progressive structural remodeling, including increased wall stiffness and altered extracellular matrix composition, which may contribute to susceptibility to aneurysmal degeneration. In the context of abdominal aortic aneurysm (AAA), wall stress represents a key determinant of aneurysm expansion and rupture risk. Interestingly, epidemiological and clinical observations indicate that patients with diabetes mellitus (DM) have a slower rate of AAA growth than non-diabetic individuals, with reported differences that are modest but consistent across studies (approximately 20–30% lower expansion rates) [27]. One proposed explanation is altered vascular remodeling in diabetes, which may confer a degree of structural stability to the aortic wall. A potential mechanism underlying this effect is the increased extracellular matrix (ECM) deposition observed in diabetes, leading to greater matrix volume and consequent thickening of the aortic wall. This interpretation is primarily based on biomechanical principles and indirect structural observations rather than on direct diabetes-specific patient data [28]. In addition, chronic hyperglycemia promotes the formation of advanced glycation end products (AGEs), which induce non-enzymatic cross-linking of collagen fibers. This process increases collagen stiffness and resistance to proteolytic degradation and may stabilize the vascular wall architecture [29]. Furthermore, hyperglycemia and the diabetic milieu may influence extracellular matrix remodeling through context-dependent changes in MMP activity and in the balance between MMPs and their tissue inhibitors (TIMPs), as well as through alterations in VSMC phenotype and function. Because VSMCs are major regulators of extracellular matrix synthesis and structural integrity, diabetes-associated changes in VSMC behavior could influence aneurysm remodeling; however, direct evidence demonstrating a diabetes-specific effect on these pathways in human AAA remains limited [14,15,30,31]. Although these mechanisms provide a plausible explanation for the inverse association between diabetes and AAA, they may also contribute to increased arterial stiffness and other adverse cardiovascular outcomes, highlighting the complex and multifaceted impact of diabetes on vascular biology.

## 4. Medical Treatment

Diabetes management includes lifestyle interventions and pharmacological therapies with distinct mechanisms of action aimed at improving glycemic control and reducing diabetes-related complications. Evidence on antidiabetic therapies and aortic aneurysm is highly heterogeneous and includes observational clinical studies, meta-analyses, animal models, and in vitro experiments. These levels of evidence should not be considered equivalent, and associations observed in treated diabetic populations may reflect both drug-specific pleiotropic effects and differences in glycemic control or underlying disease severity.

### 4.1. Metformin

Metformin is a biguanide and remains one of the most widely used oral glucose-lowering agents for the management of type 2 diabetes. Its principal actions include suppression of hepatic glucose production and improvement of peripheral insulin sensitivity. The molecular mechanisms potentially linking metformin to aneurysm biology remain incompletely defined. Most clinical evidence derives from observational cohorts and their meta-analyses. A 2024 systematic review including 11 studies and 148,589 participants reported an association between metformin use and slower AAA growth as well as fewer AAA-related events, although substantial between-study heterogeneity remained [14]. Notably, a randomized, double-blind, placebo-controlled proof-of-concept trial published in 2025 investigated metformin in non-diabetic patients with small AAA. No significant reduction in aneurysm diameter or volume progression was observed over 12 months; however, the study enrolled only 58 participants and was substantially underpowered because the planned recruitment target was not achieved [32]. A subsequent 2026 systematic review and meta-analysis similarly found that metformin use was associated with slower AAA growth and fewer AAA-related events, but most contributing studies were observational and carried a moderate risk of bias [33]. Experimental studies additionally suggest effects on inflammation, extracellular matrix remodeling, and vascular smooth muscle cell biology that may occur partly independently of glucose lowering. Metformin also activates AMP-activated protein kinase (AMPK) signaling, which may contribute to some of its pleiotropic vascular effects [34]. Overall, metformin currently has the strongest clinical evidence among antidiabetic agents studied in AAA, but available data do not yet establish a causal aneurysm-modifying effect.

### 4.2. Thiazolidinediones

Thiazolidinediones are agonists of peroxisome proliferator-activated receptors (PPARs), nuclear receptors expressed in several tissues and involved in the regulation of glucose and lipid metabolism. PPAR agonists such as rosiglitazone and pioglitazone improve insulin sensitivity and may reduce circulating triglyceride levels [35]. The use of thiazolidinediones in DM treatment has declined because of concerns about heart failure risk associated with this drug class [34]. Evidence regarding the relationship between thiazolidinediones and aortic aneurysm is limited. Pini et al. evaluated the effects of several drugs on MMP-9 expression in mesenchymal stem cells isolated from AAA specimens and found that pioglitazone reduced MMP-9 expression by approximately 50% [36]. However, this evidence is limited to an in vitro model and does not establish an aneurysm-modifying effect in patients. Moreover, the known cardiovascular safety concerns of thiazolidinediones, particularly fluid retention and heart-failure risk, substantially limit the direct translation of these findings to clinical practice.

### 4.3. Dipeptidyl Peptidase-4 (DPP-4) Inhibitors

DPP-4 inhibitors prevent the enzymatic degradation of incretin hormones, particularly GLP-1, thereby prolonging incretin activity, enhancing glucose-dependent insulin secretion, and reducing glucagon release. In experimental AAA models, alogliptin reduced AAA formation in a dose-dependent manner; this effect was associated with reduced reactive oxygen species (ROS) activity and lower MMP-2 and MMP-9 levels [37]. Sitagliptin similarly attenuated AAA development in mice and reduced MMP-2 and MMP-9 expression, limiting elastin and collagen disruption in the aortic wall [38]. These findings are predominantly derived from experimental models. Consistent with the limited clinical evidence, a recent meta-analysis of clinical studies did not demonstrate a statistically significant association between DPP-4 inhibitor use and AAA growth [33].

### 4.4. GLP-1 Receptor Agonists

GLP-1 receptor agonists (GLP-1RAs) lower glucose by enhancing glucose-dependent insulin secretion, suppressing glucagon release, and delaying gastric emptying, and they have established cardiovascular benefits in patients with type 2 diabetes. Their potential role in aortic aneurysm disease has recently attracted increasing interest. In experimental AAA models, liraglutide reduced aortic dilation, elastin degradation, leukocyte infiltration, oxidative stress, and MMP-2 and MMP-9 activity, with greater effects when treatment was initiated at earlier stages of aneurysm development [39]. More recently, a large propensity-matched retrospective study reported that GLP-1RA prescription was associated with lower rates of mortality, AAA repair, and acute abdominal aortic syndromes in patients with pre-existing unruptured AAA, both with and without type 2 diabetes [40]. However, these clinical data remain observational and do not establish an aneurysm-specific causal effect. Accordingly, prospective studies evaluating aneurysm growth and dedicated randomized trials are required before GLP-1RAs can be considered disease-modifying therapies for AAA.

### 4.5. Sulfonylureas

Sulfonylureas are widely used oral glucose-lowering agents for type 2 diabetes mellitus. They primarily stimulate insulin secretion by inhibiting ATP-sensitive potassium (K_ATP) channels in pancreatic β-cells [41]. Their potential vascular effects have also received attention. A population-based case–control study by Hsu et al., using data from Taiwan’s National Health Insurance Research Database, reported that the use of oral hypoglycemic agents—including sulfonylureas, biguanides, and thiazolidinediones—was associated with a reduced risk of aortic aneurysm (AA) development [42]. Although the biological mechanisms underlying this association remain poorly defined, recent pooled clinical evidence has strengthened the epidemiological signal. A 2026 systematic review and meta-analysis reported that sulfonylurea exposure was associated with slower AAA growth and fewer AAA-related events; however, these findings were largely derived from observational data and remain vulnerable to residual confounding [33]. Accordingly, these clinical associations should not be interpreted as evidence of a causal therapeutic effect. Pharmacological modulation of vascular K_ATP channels may influence vascular tone and VSMC proliferation; notably, gliclazide has been shown experimentally to inhibit VSMC proliferation through CaMKKβ–AMPK signaling [43,44].

### 4.6. Sodium–Glucose Cotransporter 2 (SGLT2) Inhibitors

SGLT2 inhibitors are a relatively recent class of glucose-lowering agents used in the management of type 2 diabetes mellitus. Their primary mechanism of action is inhibition of SGLT2 transporters in the proximal convoluted tubule, which reabsorb most filtered glucose. By blocking these high-capacity, low-affinity transporters, SGLT2 inhibitors promote urinary glucose excretion (glycosuria), thereby reducing plasma glucose levels through a mechanism that is independent of insulin secretion and incretin signaling pathways [45,46]. Common agents in this class include canagliflozin, dapagliflozin, and empagliflozin. Although SGLT2 expression is predominantly renal, experimental studies have also detected SGLT2 in vascular endothelial cells and vascular smooth muscle cells, including human aortic smooth muscle cells, suggesting that at least some vascular effects of SGLT2 inhibitors may occur independently of systemic glucose lowering [47,48]. This broader tissue distribution may contribute to pleiotropic effects beyond glycemic control, including anti-inflammatory and antioxidant actions as well as established cardiovascular benefits [45,49]. Preclinical studies have explored the potential role of SGLT2 inhibitors in vascular disease, including abdominal aortic aneurysm (AAA). Ortega et al. demonstrated that chronic administration of empagliflozin significantly attenuated angiotensin II (Ang II)-induced suprarenal AAA formation in apolipoprotein E-deficient (ApoE−/−) mice [50]. This effect was associated with reduced macrophage infiltration within aneurysmal lesions and downregulation of pro-inflammatory cytokines. Furthermore, empagliflozin also reduced the expression of matrix metalloproteinases (MMP-2 and MMP-9), enzymes critically involved in extracellular matrix degradation, as well as reduced atherosclerotic burden. In the same experimental model, empagliflozin was shown to suppress vascular endothelial growth factor (VEGF) expression and inhibit pathological neovascularization, while preserving elastin integrity within the aortic wall, as demonstrated by immunohistochemical analyses [50]. Complementary in vitro findings using human aortic endothelial cells revealed that empagliflozin reduced mononuclear leukocyte adhesion and decreased the production of chemokines induced by Ang II stimulation [50]. Additional in vitro work by Behnammanesh et al. indicates that canagliflozin inhibits endothelial cell proliferation and tube formation, suggesting a potential anti-angiogenic effect [51]. Together, these findings suggest that SGLT2 inhibitors can modulate key processes involved in aneurysm pathogenesis, including inflammation, extracellular matrix remodeling, and angiogenesis.

Overall, the aneurysm-specific evidence for SGLT2 inhibitors remains predominantly preclinical. Although experimental findings support anti-inflammatory, extracellular matrix-preserving, and antiangiogenic effects, clinical evidence demonstrating reduced AAA development or progression is currently insufficient. Therefore, their established cardiovascular benefits should not be extrapolated to an aneurysm-specific therapeutic effect without dedicated clinical studies. The available clinical and experimental evidence for the principal antidiabetic drug classes investigated in AAA is summarized in Table 1.

## 5. Discussion

The available evidence supports a paradoxical inverse association between diabetes mellitus and abdominal aortic aneurysm, but the strength of this association varies substantially according to the outcome considered. The most consistent human data concern AAA prevalence and growth, whereas evidence for rupture, aneurysm-related events, and thoracic aortic aneurysm is more limited and heterogeneous. Nevertheless, the available thoracic evidence points in a similar direction. A meta-analysis of observational studies reported an inverse association between diabetes and TAA, while Mendelian randomization data subsequently suggested inverse associations of genetically predicted type 2 diabetes with both TAA risk and ascending aortic diameter [9,52]. These findings remain substantially less extensive than the evidence available for AAA and should therefore be interpreted cautiously. Importantly, these observations should not be extrapolated to aortic disease as a whole, because thoracic aneurysms and acute aortic syndromes arise from distinct genetic, structural, and biomechanical substrates. Although AAA and TAA share several pathological features, including extracellular matrix remodeling, VSMC dysfunction, oxidative stress, and altered proteolytic activity, the relative importance of these processes differs substantially. AAA is typically characterized by prominent inflammatory cell infiltration, protease-mediated extracellular matrix degradation, and a strong association with smoking and traditional cardiovascular risk factors. In contrast, ascending TAA more often reflects medial degeneration and VSMC dysfunction, with alterations in TGF-β signaling and defined genetic pathways playing a particularly important role in heritable and syndromic forms. Therefore, mechanisms potentially linking diabetes to reduced extracellular matrix degradation or altered VSMC biology may be relevant to both phenotypes, whereas findings concerning inflammation, neoangiogenesis, and intraluminal thrombus are better supported in AAA. The extent to which diabetes modifies these pathways in human TAA remains largely unresolved. Mechanistically, no single pathway adequately explains the diabetes–AAA association. Among the proposed mechanisms, altered extracellular matrix remodeling, particularly AGE-mediated collagen cross-linking and modulation of MMP activity, is supported by both human and experimental evidence [10,14,15,18]. In contrast, the contribution of reduced inflammatory cell recruitment and impaired neoangiogenesis derives predominantly from experimental models and therefore remains less firmly established in human AAA. The nature of the supporting evidence also differs across experimental settings. Human evidence comes from observational cohorts and analyses of resected AAA tissue, including recent single-cell and spatial profiling, which support alterations in extracellular matrix remodeling and vascular and immune cell states but cannot establish causality. Animal models provide more direct experimental evidence that hyperglycemia can attenuate aneurysm growth, macrophage infiltration, proteolytic activity, and neovascularization, although these effects remain model-dependent. In vitro studies further support cell-specific effects on MMP production and vascular cell behavior. These complementary approaches strengthen biological plausibility but should not be considered equivalent or directly interchangeable with findings in human AAA. Altered extracellular matrix turnover, AGE-mediated collagen cross-linking, modulation of MMP activity, changes in VSMC phenotype, inflammatory cell heterogeneity, impaired neoangiogenesis, and changes in wall biomechanics may all contribute, but their relative importance remains uncertain and is likely to vary across disease stages and experimental models. Moreover, several mechanisms that may theoretically limit aneurysm expansion, such as increased collagen cross-linking and arterial stiffness, are simultaneously associated with adverse cardiovascular consequences. The biological effects of diabetes on the aortic wall should therefore be considered context-dependent rather than uniformly beneficial.

Antidiabetic therapies add a further layer of complexity. Metformin currently has the strongest clinical evidence, although most supporting studies remain observational and the available randomized evidence is limited. For other drug classes, including DPP-4 inhibitors, GLP-1 receptor agonists, sulfonylureas, thiazolidinediones, and SGLT2 inhibitors, the balance of evidence varies from observational associations to predominantly preclinical findings. Consequently, it remains difficult to disentangle effects attributable to diabetes itself, glycemic control, and drug-specific pleiotropic mechanisms.

The apparent diabetes–aneurysm association is also likely to vary across aneurysm phenotypes. AAA biology differs according to sex, aneurysm diameter and stage, intraluminal thrombus burden, smoking exposure, inflammatory activity, and genetic background, while thoracic aneurysms arise from substantially different biological substrates. Sex is particularly relevant because AAA is more prevalent in men, whereas women may experience rupture at smaller diameters and exhibit distinct structural and biomechanical characteristics. In addition, the extent and composition of intraluminal thrombus can influence local hypoxia, inflammatory activity, proteolysis, and wall remodeling. These sources of heterogeneity mean that a diabetes-associated effect observed in one AAA population may not apply across all aneurysm phenotypes [53,54,55].

An important issue in interpreting the epidemiological association between diabetes and AAA is residual confounding. Most available human studies are observational, and diabetic and non-diabetic populations may differ substantially in smoking exposure, use of statins and other cardiovascular medications, renal function, obesity, metabolic profile, healthcare utilization, and intensity of imaging surveillance. Antidiabetic treatment represents an additional source of confounding, because part of the observed association may reflect drug exposure rather than diabetes itself. Competing mortality may also influence observational estimates, as patients with diabetes have a higher burden of cardiovascular and non-cardiovascular disease and may die before an aneurysm is diagnosed, progresses, or reaches a clinically relevant endpoint. Furthermore, definitions of diabetes, AAA, and aneurysm growth, as well as imaging modalities and surveillance intervals, vary considerably across studies. These factors limit causal inference even when multivariable adjustment is performed and may partly explain the heterogeneity observed across contemporary meta-analyses [7,33,56].

Overall, the current literature supports a clinically intriguing but incompletely explained association rather than a proven protective effect of diabetes. The predominance of observational clinical evidence, together with substantial heterogeneity in diabetic phenotypes, aneurysm characteristics, treatment exposure, and experimental models, limits causal inference. Future studies should combine longitudinal clinical cohorts with molecular profiling, spatial and single-cell approaches, and patient-specific biomechanical assessment to determine which components of the diabetes-associated phenotype are genuinely relevant to aneurysm progression and whether any of these pathways can be targeted safely without reproducing the adverse systemic consequences of diabetes.

## 6. Conclusions

Current evidence supports an inverse association between diabetes mellitus and the development and progression of aortic aneurysms, particularly AAA. This association is best established for AAA, whereas evidence concerning TAA and aortic dissection remains substantially less robust and should not be extrapolated from the abdominal phenotype. Potential mechanisms include reduced extracellular matrix degradation, increased collagen cross-linking, modulation of inflammatory responses, and attenuation of pathological neoangiogenesis. Antidiabetic therapies, particularly metformin, may contribute to this association, although their independent effects remain incompletely defined. Diabetes-associated changes in aortic wall structure and biomechanics may also influence aneurysm progression.

However, the relationship between diabetes and aortic disease remains complex and not fully understood. While an inverse association with aneurysm progression has been consistently reported, this association coexists with broader adverse cardiovascular consequences of diabetes.

Diabetes is associated with substantial morbidity and mortality and therefore cannot be considered clinically beneficial. Nevertheless, certain pathophysiological and molecular features of diabetes, together with the effects of antidiabetic therapies, may contribute to the observed inverse association with aortic aneurysm development. Investigating this intriguing association may provide novel insights into the pathophysiology of aneurysmal disease and, ultimately, contribute to a more complete understanding of the molecular mechanisms underlying aneurysm formation and progression. Further translational and clinical research is required to clarify these mechanisms and to determine whether targeting diabetes-related pathways could represent a novel strategy for the prevention and treatment of aortic aneurysms.

## 7. Future Perspectives

Future research should focus on elucidating the mechanistic relationship between DM and aortic aneurysm disease through integrated translational approaches combining molecular biology, vascular biomechanics, and clinical investigation. Although current evidence supports an inverse association between DM and AAA progression, the precise biological pathways underlying this phenomenon remain incompletely understood. In particular, further studies are needed to clarify how chronic hyperglycemia, AGEs, ECM remodeling, and inflammatory modulation interact across different stages of aneurysm development. A major research priority will be the identification of specific molecular targets involved in the diabetes-associated modulation of MMP activity and vascular wall degradation. Better characterization of pathways involving RAGE, oxidative stress, vascular smooth muscle cell phenotypic modulation, and macrophage polarization may provide novel therapeutic opportunities aimed at stabilizing aneurysm growth independently of glycemic status. In parallel, advances in imaging technologies and computational biomechanics may improve understanding of the effects of diabetes on aortic wall stress, arterial stiffness, and rupture risk. Future studies integrating finite element analysis, wall shear stress assessment, and tissue composition imaging could facilitate the identification of biomechanical markers associated with aneurysm stability in diabetic patients. Such approaches may also contribute to the development of more personalized risk stratification models extending beyond aneurysm diameter alone. Another important area of investigation concerns the vascular effects of antidiabetic therapies. Metformin is currently the most extensively studied agent; however, growing experimental evidence suggests that DPP-4 inhibitors, GLP-1 receptor agonists, and SGLT2 inhibitors may exert anti-inflammatory, antiproteolytic, and antiangiogenic effects within the aortic wall. Large prospective randomized clinical trials are therefore needed to determine whether these agents can reduce aneurysm growth rates or rupture risk in both diabetic and non-diabetic populations. In this context, drug repurposing strategies may represent a clinically relevant approach to aneurysm management. Future investigations should also address the intrinsic heterogeneity of diabetes itself. Differences between type 1 and type 2 diabetes, disease duration, glycemic control, insulin resistance, and associated metabolic conditions may influence aneurysm biology through distinct mechanisms. Stratified analyses based on metabolic phenotype and therapeutic exposure will therefore be important for better defining the clinical implications of the diabetes–aneurysm relationship. Finally, multicenter longitudinal studies integrating genomic, proteomic, and metabolomic approaches may facilitate the identification of novel biomarkers predictive of aneurysm progression and therapeutic response.

A deeper understanding of the paradoxical inverse association between DM and aortic aneurysm disease may ultimately support the development of therapies directed at diabetes-associated vascular pathways that may be relevant to reduced AAA progression, without reproducing the systemic complications of chronic hyperglycemia.

## Figures and Tables

**Table 1:** Evidence supporting potential effects of antidiabetic therapies in abdominal aortic aneurysms.

Drug Class	Evidence Type	Population/Model	Main Findings	Proposed Mechanisms	Main Limitations
Metformin	Observational studies, meta-analyses, small RCT, preclinical	Human AAA Experimental AAA	Associated with slower AAA growth and fewer AAA-related events; small RCT showed no significant benefit	Anti-inflammatory effects; ECM/MMP and VSMC modulation; AMPK-related pathways	Predominantly observational evidence; residual confounding; RCT underpowered
Thiazolidinediones (TZDs)	Mainly in vitro/ex vivo	AAA-derived human cells	Pioglitazone reduced MMP-9 expression	PPAR-γ activation; MMP and ECM modulation	Very limited AAA-specific evidence; heart-failure safety concerns
DPP-4 inhibitors	Mainly preclinical; limited clinical data	Experimental AAA Observational human data	Alogliptin and sitagliptin attenuated experimental AAA; no significant clinical effect on AAA growth	Enhanced GLP-1 signaling; reduced oxidative stress and MMP activity; monocyte modulation	Predominantly preclinical evidence; no dedicated RCTs
GLP-1 receptor agonists	Animal studies; retrospective human data	Experimental AAA Patients with unruptured AAA	Liraglutide attenuated experimental AAA; human studies suggest fewer AAA-related events	Anti-inflammatory and antioxidant effects; reduced MMP activity and leukocyte infiltration	Human evidence observational; no AAA-specific RCT
Sulfonylureas	Observational studies; mechanistic studies	Diabetic AAA populations Vascular smooth muscle cells	Associated with slower AAA growth and fewer AAA-related events; gliclazide inhibits VSMC proliferation experimentally	KATP-channel and VSMC modulation	Residual confounding; limited AAA-specific mechanistic evidence
SGLT2 inhibitors	Mainly animal and in vitro	Experimental AAA Vascular cells	Reduced experimental AAA formation, inflammation, MMP expression and neovascularization	Anti-inflammatory, antioxidant and antiangiogenic effects	Insufficient AAA-specific human evidence

## Data Availability

No new data were created or analyzed in this study. Data sharing is not applicable to this article.

## Abbreviations

The following abbreviations are used in this manuscript:

TAA	Thoracic aortic aneurysms
AAA	abdominal aortic aneurysms
AA	aortic aneurysms
DM	diabetes mellitus
T2DM	type 2 diabetes mellitus
MMP	matrix metalloproteinases
AGE	advanced glycation end product
ECM	extracellular matrix
NF-κB	nuclear factor kappa B
ILT	intraluminal thrombus
VEGF	vascular endothelial growth factor
RAGE	receptor for advanced glycation end products
PPAR	peroxisome proliferator-activated receptor
DPP-4	dipeptidyl peptidase-4
GLP-1	glucagon-like peptide-1
ROS	reactive oxygen species
K-ATP	ATP-sensitive potassium
SUR	sulfonylurea receptor subtype
VSMC	vascular smooth muscle cells
SGLT2	sodium–glucose cotransporter 2
Ang II	angiotensin II
ApoE	apolipoprotein E
